# 3-*O*-trans-*p*-coumaroyl esterification enhances the anti-inflammatory effects of tormentic acid by targeting NF-κB signaling

**DOI:** 10.1016/j.redox.2025.103731

**Published:** 2025-06-14

**Authors:** Mara Heckmann, Lea Karlsberger, Bernhard Blank-Landeshammer, Gerald Klanert, Nadiia Sadova, Verena Stadlbauer, Georg Sandner, Theresa Gramatte, Simone Kasis, Julian Weghuber

**Affiliations:** a Center of Excellence Food Technology and Nutrition, University of Applied Sciences Upper Austria, Stelzhamerstraße 23, 4600, Wels, Austria; b FFoQSI GmbH–Austrian Competence Centre for Feed and Food Quality, Safety and Innovation, Technopark 1D, Tulln, 3430, Austria

**Keywords:** Pentacyclic triterpene, Tormentic acid, Hydroxycinnamoyl esterification, NF-κB, Pro-inflammatory signaling

## Abstract

Tormentic acid (TA), a plant-derived pentacyclic triterpene, exhibits antioxidant and anti-inflammatory potential, yet the pharmacological effects of its 3-*O*-trans-*p*-coumaroyl ester (*trans*-TACE) remain underexplored. This study investigates how hydroxycinnamoyl esterification influences the biological activity of pentacyclic triterpenes by comparing TA and *trans*-TACE in cellular and *in vivo* stress models.

We assessed their ability to mitigate oxidative stress by evaluating the inhibition of ROS and NO molecules. Pro-inflammatory cytokine production in LPS-stimulated THP-1 macrophages was analyzed through cytokine arrays and multiplex immunoassays, while NF-κB activation was examined in both TLR4-dependent and -independent models using HEK-Blue reporter cells. Uptake efficiencies into Caco-2 enterocytes were measured via LC-MS. The *in vivo* relevance of these findings was assessed using *C. elegans* as a model for oxidative and inflammatory stress response.

Results showed that *trans*-TACE significantly reduced cellular ROS and NO levels compared to TA. Protein analyses of LPS-stimulated THP-1 macrophages indicated that *trans*-TACE significantly decreased pro-inflammatory cytokines involved in NF-κB signaling (*e.g*., TNFα, IL-8, CCL2, CXCL5 and CXCL11). *Trans*-TACE also inhibited NF-κB activation in both TLR4-dependent and -independent models. In *C. elegans*, both TA and *trans*-TACE downregulated several stress-induced genes, with *trans*-TACE exhibiting broader effects by additionally targeting *daf-16* and *gst-4* gene expression. Moreover, we revealed key differences in bioactivities between the trans and cis isoform of TACE, underscoring the importance of considering the structural properties of geometric isomers in therapeutic assessments.

Overall, this study suggests that esterification significantly enhances the biological activity of pentacyclic triterpenes and points towards new possibilities for developing effective natural anti-inflammatory therapies.

## Highlights

• Esterification enhances the bioactivity of the pentacyclic triterpene TA.
• The derivative TACE suppresses both TLR4-dependent and -independent NF-κB activation.
• TACE inhibits a broad spectrum of pro-inflammatory cytokines and chemokines *in vitro.*
• *C. elegans* data support TACE's role in stress adaptation and immune regulation.
• TACE's activity in targeting NF-κB signaling is isomer-specific.

## Graphical abstract

**Figure:**
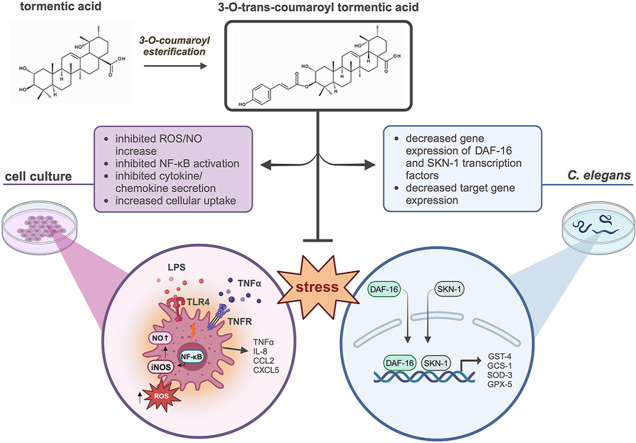

## 1 Introduction

Oxidative stress and inflammation are closely linked to the pathogenesis of numerous chronic diseases, including atherosclerosis, diabetes, neurodegenerative disorders, and cancer [1]. Oxidative stress arises from an imbalance between the production of reactive oxygen species (ROS) and the intrinsic antioxidant defenses, leading to cellular damage. The transcription factor nuclear factor kappa B (NF-κB) plays a crucial role in regulating immune response, inflammation, and cellular stress. Activation of NF-κB triggers the production of pro-inflammatory cytokines, chemokines, and other mediators that perpetuate inflammation and oxidative stress [2]. Chronic inflammation, often driven by oxidative stress, involves the continuous activation of inflammatory pathways, contributing to tissue damage and disease progression. Thus, compounds that can modulate inflammation and reduce NF-κB activation are of significant interest for their potential to combat chronic inflammation and its associated diseases [3].

Plants and their bioactive compounds have been widely recognized as valuable sources of novel therapeutic agents for the prevention and treatment of a wide range of diseases [4]. Pentacyclic triterpenes represent a large family of secondary plant metabolites, characterized by a high diversity of structures and bioactive properties. These compounds are typically found on the surfaces of plants, such as stem barks [5] or leaves, and they accumulate significantly in the fruit peels of apples and pears [6]. Pentacyclic triterpenes are composed of six isoprene units and are divided into three subclasses: the ursane, oleanane, and lupane series, each comprising molecules with important bioactive properties [7].

**Fig. 1:**
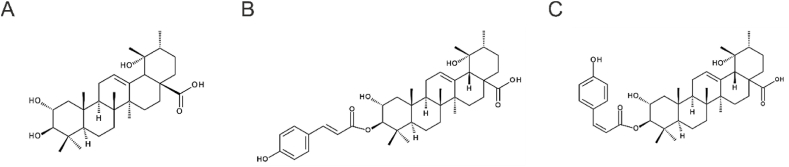
Chemical structures of tormentic acid (A) and its *trans*- (B) and *cis*- (C) hydroxycinnamoyl esters at the C3 position.

Tormentic acid (TA, Fig. 1A) belongs to the ursane subclass of pentacyclic triterpenes and is predominantly found in the Rosaceae family, especially in the leaves, but also in the Lamiaceae and Urticaceae families, where it plays a role in plant defense against pathogens. TA is known for its metabolism-regulating, cytoprotective and anti-inflammatory effects [7]. It lowers the fasting blood glucose level [8] through the release of glucose-dependent insulin [9] and prevents carbohydrate breakdown by inhibiting alpha-glucosidase [10,11]. Although TA has not shown free radical scavenging activity [12,13], it activated the antioxidant defense system in the liver of mice, thereby protecting against induced hepatotoxicity and oxidative stress [[14], [15], [16]]. TA inhibits NF-κB and mitogen-activated protein kinase (MAPK) signaling, which is thought to reduce nitric oxide (NO) and Prostaglandin E2 production by lowering the levels of nitric oxide synthase 2 and prostaglandin-endoperoxide synthase 2. However, other *in vitro* studies have shown inconsistent results regarding TA's impact on NO generation, ranging from low [17] to no effect [[18], [19], [20]]. Additionally, reduced serum levels of tumor necrosis factor alpha (TNFα), interleukin (IL)-1β and IL-6 were observed, all of which are mainly known as pro-inflammatory signaling molecules [[14], [15], [16]]. The reduction of NF-κB signaling and pro-inflammatory factor release was also observed in an Alzheimer's disease model in mice [21].

In nature, pentacyclic triterpenes can also form pentacyclic triterpene hydroxycinnamoyl esters at the C3 position, which have been less extensively studied. These ester derivatives have demonstrated promising potential, as modifications at this position can enhance bioavailability, solubility, and biological activity compared to their non-esterified counterparts [[22], [23], [24]]. One such derivative of TA, 3-*O*-*p*-coumaroyltormentic acid (TACE), has demonstrated promising bioactive potential in some recent studies, yet it remains largely underexplored. TACE isolated from *Eriobotrya japonica* leaves has demonstrated anti-leukemia properties by exhibiting apoptotic and cytotoxic activities in the human leukemia cell line HL60 [25]. Recently, TACE was also identified in *Vitellaria paradoxa* leaves where its anti-trypanosoma activity was highlighted in a recent patent [26]. While in many studies anti-inflammatory effects of extracts containing TA and purified TA were investigated, data on the anti-inflammatory potential of TACE are scarce. It has been demonstrated that TACE can reduce ear and paw edema [27] and inhibit lipopolysaccharide (LPS)-induced NO generation [18]. This lack of data indicates a need for deeper insight into the anti-inflammatory and antioxidant potential of TACE in direct comparison to TA.

Moreover, hydroxycinnamoyl esters of pentacyclic triterpenes can exist as geometric isomers, with potential differences in their bioactivity [22,23]. TACE naturally occurs as a mixture of trans and cis isomers (Fig. 1B and C), yet whether this structural variation affects its biological activity remains unclear [28]. Most studies on TACE have focused on the trans isoform, but a direct comparison of the cis and trans isoforms has been lacking. Identifying the functional relevance of geometric isomerism is crucial for accurately evaluating the bioactivity of TACE and similar compounds, as even subtle structural differences can significantly impact their therapeutic potential.

In this study, we aimed to compare the biological efficacy of TA and TACE, focusing on their antioxidant and anti-inflammatory properties using various *in vitro* and *in vivo* models of oxidative and inflammatory stress. Specifically, we examined their ability to inhibit NF-κB activation, regulate pro-inflammatory cytokines and chemokines, and modulate key cellular stress-related pathways. Additionally, we investigated whether hydroxycinnamoyl esterification and geometric isomerism influence the bioactivity of TACE. By selecting TA as a model compound, we aimed to provide broader insights into how esterification at the C3 position and the resulting structural modifications affect the therapeutic potential of pentacyclic triterpenes, ultimately contributing to the development of more effective natural therapies targeting inflammation and oxidative stress.

## 2 Material and methods

### 2.1 Cell culture

RAW264.7 mouse macrophage-like (ATCC, Manassas, VA, USA), Caco-2 human epithelial and monocytic THP-1 (DSMZ, Braunschweig, Germany) cell lines were cultured under conditions of 37 °C, 5 % CO_2_ and humidified air in Dulbecco's Modified Eagle Medium (DMEM, +4.5 g/l glucose, + stable glutamine, + sodium pyruvate. + 3.7 g/l sodium bicarbonate), Eagle Minimum Essential Medium (EMEM, + earle's balanced salts, +2 mM L-glutamine, + non-essential amino acid, +1 mM sodium pyruvate, +1.5 g/l sodium bicarbonate) and Roswell Park Memorial Institute 1640 Medium (RPMI 1640, +2 mM L-glutamine + 1 mM sodium pyruvate + 10 mM HEPES + 4.5 g/l glucose + 1.5 g/l sodium bicarbonate + 0.05 μM 2-mercaptoethanol), respectively, supplemented with 10 % fetal bovine serum (FBS) and 100 U/ml-100 μg/ml penicillin-streptomycin (P–S) (all from PAN-Biotech, Aidenbach, Germany). The cells were subcultured two times a week. To induce differentiation into a mature macrophage-like state, the THP-1 monocytes were treated with 50 ng/ml phorbol 12-myristate 13-acetate (PMA, Sigma Aldrich, St. Louis, MO, USA) for 48 h. Consequently, the suspended monocytes were differentiated into adherent macrophages. Successful differentiation was defined by an adherence rate of ∼90 %. All cell culture experiments with the THP-1 cell line were performed in differentiated THP-1 cells.

The HEK-Blue hTLR4 reporter cell line, which stably co-expresses an NF-κB-inducible secreted embryonic alkaline phosphatase (SEAP) reporter gene and the human toll like receptor (TLR) 4 gene, along with its corresponding parental cell line, HEK-Blue null2, which only expresses the SEAP reporter gene, were obtained from InvivoGen (San Diego, CA, USA). These cell lines were cultured in DMEM containing 4.5 g/l glucose, stable glutamine, sodium pyruvate, and 3.7 g/l sodium bicarbonate under conditions of 37 °C, 5 % CO_2_ and humidified air. The medium was supplemented with 10 % heat-inactivated FBS, 100 U/ml-100 μg/ml P–S and 100 μg/ml Normocin™ (InvivoGen), as well as cell line-specific antibiotics 1X HEK-Blue Selection for the HEK-Blue hTLR4 cell line and 100 μg/ml Zeocin for the HEK-Blue null2 cell line (both from InvivoGen).

Cell culture experiments were performed at a maximum passage number of 35 for Caco-2, THP-1 and RAW264.7 cells and of 20 for HEK-Blue reporter cells.

### 2.2 Treatment compounds

Tormentic acid (TA) with a purity of ≥95 % was obtained from Sigma Aldrich, whereas purified 3-*O*-trans-*p*-coumaroyltormentic acid (*trans*-TACE, purity ≥95 %) was kindly provided by Dr. Legay from the Luxembourg Institute of Science and Technology (LIST). The compound was produced and purified according to a patented process involving the biotechnological production of 3-*O*-trans-*p*-coumaroyltormentic acid from plant cell suspension cultures [26]. For additional experiments, 3-*O*-trans/*cis*-*p*-coumaroyltormentic acid (trans/*cis*-TACE), with a trans/cis ratio of approximately 4:6, and 3-*O*-cis-*p*-coumaroyltormentic acid (*cis*-TACE) with purity ≥95 % were obtained from Novachemistry (Loughborough, UK).

To analyze the trans and cis isomer content in the TACE samples, isomers were separated using an Ultimate 3000 HPLC system equipped with a built-in degasser, a binary pump, autosampler, heated column-compartment and a diode-array detector (DAD, all Thermo Fisher Scientific, Waltham, MA, USA). An Accucore C18 column (150 mm × 2.1 mm inner diameter, 2.6 μm particle size, Thermo Fisher Scientific) kept at 25 °C was employed for separation under isocratic conditions. A mixture of 50 % (v/v) water and acetonitrile (each with 0.1 % formic acid) at a flow rate of 0.5 ml/min was used with a runtime of 15 min to achieve baseline separation of the cis and trans isomers. Injection volume was 4 μl and the DAD was set to a wavelength of 324 nm for detection. The system was operated using Chromeleon 7.2.10 software.

Cytotoxicity of the treatment compounds was assessed following 1.5 h of incubation with 50 μM resazurin sodium salt solution (Sigma Aldrich) using a POLARstar Omega microplate reader (BMG Labtech, Ortenberg, Germany) in fluorescence mode at 544 nm excitation and 590 nm emission wavelengths. Only non-cytotoxic concentrations (cell viability >90 %) were used in all subsequent cell culture experiments (see Supplementary Table 1). For supplementary experiments, dehydroxymethylepoxyquinomicin (DHMEQ) (≥98 % purity) was obtained from Sigma Aldrich.

### 2.3 Determination of uptake efficiencies in differentiated Caco-2 cells

For compound uptake studies, Caco-2 cells were seeded in triplicate into 6-well plates at a concentration of 2 × 10^6^ cells/well and grown overnight. The cells were then differentiated using DMEM supplemented with 100 U/ml-100 μg/ml P–S, 0.1 % MITO + Serum Extender (Corning Inc., Corning, NY, USA) and 5 mM butyric acid (Sigma Aldrich). This medium was refreshed on the following day. Differentiation was confirmed by the formation of domes under the microscope [29]. Differentiated Caco-2 cells were washed with fasted state simulated intestinal fluid (FaSSIF) [30], prepared according to Ilardia-Arana et al. [31], with final concentrations of 3 mM taurocholic acid (Sigma Aldrich) and 0.75 mM L-α-lecithin (Merck KGaA, Darmstadt, Germany) in Hank's Balanced Salt Solution (HBSS; Pan Biotech), adjusted to a pH of 6.5. The cells were then treated with 10 μM of the treatment compounds diluted in FaSSIF (2 ml/well) and incubated for 4 h at 37 °C. The cell layers were washed with FaSSIF and then treated with 1 ml/well of a 3:2 hexane-isopropanol solution (Honeywell Austria GmbH, Linz, Austria) on a plate shaker for 10 min. This extraction step was repeated once. The extracts were collected and dried using a Vacuum Concentrator (Labconco, Kansas City, USA) at 45 °C for 1 h. For HPLC measurements, the dried extracts were dissolved in 50 % isopropanol containing 1 % formic acid. Following the extraction, the cells were dried at room temperature for 10 min and lysed with 2 ml/well of 0.1 M sodium hydroxide on a plate shaker for 1 h. The total protein concentration in each well was determined using the Micro BCA™ Protein Assay Kit (Thermo Fisher Scientific) according to the manufacturer's instructions for normalization to the protein content. Results were normalized to the amount of the compounds in the treatment solution and to the total protein content in each replicate.

#### 2.3.1 HPLC analysis

The treatment compounds were quantified in the cell culture supernatant and the cell extract by LC-MS. A Vanquish Flex UHPLC system was used, equipped with a built-in degasser, a binary pump, autosampler, heated column-compartment, coupled to an ISQ-EC mass spectrometer via a HESI ion-source (all Thermo Fisher Scientific). Chromatographic separation was achieved using an Accucore C18 column (150 mm × 2.1 mm inner diameter, 2.6 μm particle size, Thermo Fisher Scientific) heated to 60 °C and an injection volume of 2 μl. Gradient elution was performed at a flow rate of 0.5 ml/min with mobile phase A (50 % isopropanol, 5 mM ammonium formate, pH 3.65) and B (isopropanol, 5 mM ammonium formate, pH 3.65), starting at 99.9 % A and 0.1 % B, then increasing to 99.9 % B within 5 min and a hold-time of 2 min at 99.9 % B. Finally, B was reduced to 0.1 % again and the column was equilibrated for 3 min prior to the next injection. The mass spectrometer was operated in negative ion mode and the HESI source was set to a voltage of −2.5 kV with the vaporizer heated to 280 °C and the transfer tube to 250 °C. Sheath gas pressure was set to 35 psig, Aux gas to 8 psig and Sweep gas to 1 psig. Spectra were recorded in single ion monitoring (SIM) mode with the quadrupole set to 487.3 *m/z* and 633.4 *m/z* (deprotonated TA and *trans*-TACE, [M − H]^-^), an isolation width of 0.1 *m/z* and a dwell time of 0.05 s. Source CID voltage was set to 20 V in order to reduce formation of formic acid adducts.

### 2.4 Detection of ROS production in Caco-2 and THP-1 cells

Intracellular ROS production was quantified using the cell-permeant probe H_2_DCF-DA following stress induction by the free-radical-generating compound 2,2′-azobis (2-amidinopropane) dihydrochloride (AAPH) in Caco-2 and THP-1 cells. Upon entering the cell, H_2_DCF-DA is enzymatically cleaved and, in the presence of ROS, oxidized to produce the highly fluorescent 2′,7′-dichlorofluorescein (DCF). The fluorescence intensity increase is directly proportional to the amount of ROS generated, allowing for quantification of intracellular ROS levels [32]. DCF fluorescence was quantified as described [33] with minor modifications.

The cells were seeded in triplicate into 96-well plates (Greiner Bio-One GmbH, Kremsmünster, Austria) at a density of 1 × 10^5^ cells/well and incubated overnight. For THP-1 cells a prior differentiation step was conducted by adding 50 ng/ml PMA for 48 h. The cells were then co-treated with 100 μl of 50 μM H_2_DCF-DA (Sigma Aldrich) and 10 μM of the treatment compounds at 37 °C for 20 min. An antioxidant positive control using 10 μM quercetin (Sigma Aldrich) was included in each experiment. After treatment, cells were washed with HBSS and then treated with 100 μl of 300 μM AAPH (Sigma Aldrich) in HBSS or HBSS alone as a control. The production of DCF was measured using a microplate reader (POLARstar Omega, BMG Labtech) in fluorescence mode with excitation at 485 nm and emission at 530 nm. Measurements were taken immediately after AAPH addition and every 30 min for 1.5 h. The DCF fluorescence intensity was background corrected, and area under the curve (AUC) values based on the four different measurement timepoints, assuming a linear relationship between the measurements, were calculated and normalized to the AAPH stress control.

### 2.5 Detection of NO production in RAW264.7 cells

NO production in RAW264.7 macrophages was assessed after LPS-stimulation and treatment with the compounds. This assay detects nitrite (NO_2_−) in the culture medium, which reacts with sulfanilamide and N-(1-naphthyl) ethylenediamine dihydrochloride (NED) to produce a colored azo compound. The color intensity, measured spectrophotometrically, is directly proportional to the concentration of NO_2_−, a stable byproduct of NO [34]. NO detection was performed using the Promega Griess Reagent System (Promega Corporation, Fitchburg, WI, USA) as per the manufacturer's protocol with minor modifications as previously described [35]. Briefly, RAW264.7 macrophages were seeded in triplicate in 96-well plates (Greiner Bio-One GmbH) at a density of 6.4 × 10^4^ cells/well and incubated overnight at 37 °C. The cells were then treated with 250 ng/ml LPS (Sigma Aldrich) to induce NO production, or LPS combined with 10 μM of the treatment compounds or 20 μM quercetin as a positive control. After a 24-h incubation at 37 °C, 5 % CO_2_ and humidified air, 50 μl of sulfanilamide solution were added to 50 μl of each cell supernatant or 50 μl of a nitrite standard. The plate was incubated for 10 min at room temperature. Subsequently, 50 μl of NED solution were added and incubated for an additional 10 min. Absorbance at 548 nm was measured using the POLARstar Omega microplate reader and NO_2_− levels of the samples were quantified with the standard. Results were background-corrected and normalized against the LPS stress control.

### 2.6 Determination of pro-inflammatory cytokine production in THP-1 cells

THP-1 cells were seeded in duplicate into 6-well plates at a density of 2.7 × 10^6^ cells/well and treated with 50 ng/ml PMA for 48 h to induce differentiation. The treatment compounds were diluted in medium without FBS to a final concentration of 10 μM, and 250 ng/ml LPS were added as a stressor. The cells were washed with phosphate buffered saline (PBS, PAN-Biotech) and treated with 2.5 ml of the dilutions and incubated for an additional 4 or 24 h. Then, supernatants were collected, centrifuged at 200 g for 4 min and stored at −80 °C for subsequent cytokine secretion analysis.

#### 2.6.1 Cytokine multiplex immunoassay

The secretion of certain inflammatory cytokines and chemokines, including TNFα, IL-8, CC-chemokine ligand (CCL) 2, C-X-C motif chemokine ligand (CXCL) 5 and CXCL11, into the supernatant of LPS-stimulated THP-1 cells was quantified using a custom 5-plex Luminex xMAP® assay (Bio-Techne Ltd., Abingdon, UK) following the manufacturer's protocol. Cell culture supernatants were used undiluted and diluted 1:100. Briefly, 50 μl of each sample or standard were incubated with 50 μl of precoated MagPlex microbeads for 2 h. After incubation, the beads were washed and coated with 50 μl of biotinylated antibodies. Following a 1-h incubation, the beads were washed again and treated with Streptavidin-PE solution for 30 min. The beads were then washed once more and resuspended in 100 μl of washing buffer. All incubation steps were performed in a sealed, light-protected, black-bottom plate using a plate shaker at 800 rpm. The prepared samples were measured using the Luminex® 200™ system and analyzed with xPonent® acquisition software, version 4.3 (both Luminex Corp., Austin, TX, USA).

#### 2.6.2 Cytokine array analysis

To assess the cytokine, chemokine, and growth factor expression profile, the semi-quantitative Human XL Cytokine Array Kit (Proteome Profiler™ Array, Bio-Techne Ltd.) was utilized following the manufacturer's guidelines. Briefly, membranes were blocked at room temperature for 60 min, then incubated with samples overnight at 4 °C. Following thorough washing steps, the membranes were exposed to biotinylated detection antibodies at room temperature for 90 min. Subsequent treatment involved incubation with streptavidin-horseradish peroxidase at room temperature for 30 min. To visualize the captured proteins, we employed chemiluminescent detection reagents for 1 min, and luminescence was quantified using a ChemiDoc MP Imaging System (Bio-Rad Laboratories, Hercules, CA, USA). For analysis, comparisons between treatments were conducted utilizing Image Lab Software (Bio-Rad Laboratories). Each spot's pixel intensity value was loaded into R (R Foundation for Statistical Computing, Vienna, Austria), and a global background subtraction was performed. Different timepoints of each treatment and molecule were compared for significant differences as described in 2.10. As none of the signals were significantly different between the two tested timepoints for each treatment and molecule, data from the different timepoints were combined. Molecules where all average signals per treatment were below the limit of detection (LOD; < global background + 3 × standard deviation (SD) of global background) were removed from the data set and marked as not detected. The SD and coefficient of variation (CV) of each molecule and treatment were calculated and trimmed (highest and lowest 5 % of values were removed). Global SD and global CV were calculated. Array spots for molecules and treatments that had a SD higher than the global SD and a CV higher than the global CV were visually inspected, and spot signals that were influenced by highly expressed neighboring molecules were excluded from further analysis. Again, molecules with all treatments below the LOD were removed and marked as not detected. Statistical testing was done as described in 2.10. to detect molecules with significant differences between treatments. Log_2_ fold changes were calculated for the comparisons of different treatments and loaded into Cytoscape software platform (Institute for Systems Biology, Seattle, WA, USA, version 3.10.1) for visualization.

### 2.7 Determination of NF-κB activation in HEK-Blue cells

To determine NF-κB activation following treatment with LPS, TNFα, and the treatment compounds, the HEK-Blue hTLR4 reporter cell line and its parental cell line HEK-Blue null2 were utilized by measuring the amount of secreted downstream target SEAP. The inclusion of the parental cell line allows for assumptions regarding the involvement of the TLR4 receptor. Briefly, 20 μl of LPS, TNFα and the treatment substances with or without LPS and TNFα diluted in DMEM (+10 % heat-inactivated FBS, +100 U/ml-100 μg/ml P–S, +100 μg/ml Normocin™) with final concentrations of 10 ng/ml LPS or TNFα and 5 μM of the treatment compounds were transferred to 96-well plates. 180 μl of HEK-Blue hTLR4 cells and HEK-Blue null2 cells were then seeded in triplicate into the 96-well plates containing the treatment solutions at 1 × 10^5^ and 0.6 × 10^5^ cells/well, respectively. After incubation for 24 h at 37 °C, 20 μl of the cell culture supernatants were mixed with 180 μl of QUANTI-Blue (InvivoGen) solution containing the SEAP substrate. Absorbance was measured at 620 nm following 30 min of incubation at 37 °C using the POLARstar Omega microplate reader. Viability of cells was confirmed using the CellTiter-Glo® Cell Viability Assay (Promega Corporation) according to the supplier's instructions. Results were background-corrected and normalized to the unstressed control.

### 2.8 Molecular docking studies

*In silico* analyses were carried out using targeted molecular docking with AutoDock Vina (Scripps Research Institute) [36], implemented via UCSF Chimera v1.18 [37]. NF-κB (p50/p65) was selected as the receptor, while *trans*-TACE, *cis*-TACE and the known direct NF-κB inhibitor DHMEQ [38] served as ligands. The receptor's 3D structure was sourced from the Protein Data Bank (PDB ID: 1VKX) [39], and ligand structures for TA (PubChem CID 73193) [40], *trans*-TACE (PubChem CID 68210719) [41], *cis*-TACE (PubChem CID 102004779) [42] and DHMEQ (PubChem CID 9881652) [43] were obtained from the PubChem-NCBI database. Prior to docking, the NF-κB receptor structure was prepared by removing the DNA chain, water molecules, and any nonstandard residues, followed by addition of hydrogens, charge merging, and the elimination of non-polar hydrogens. Ligands underwent similar charge merging and non-polar hydrogen removal. Blind docking was applied, with the docking grid encompassing the entire receptor, centered at x = −0.106, y = 35.969, and z = 58.276, and grid dimensions set to 56.85 Å × 91.176 Å × 102.746 Å. AutoDock Vina parameters included an exhaustiveness of 8, a maximum energy difference of 3 kcal/mol, and generated 9 binding modes. For each ligand, the binding mode with the lowest estimated free energy change (ΔG) upon binding was selected as the most thermodynamically favorable for further interaction analysis and visualized using BIOVIA Discovery Studio 2024 (Dassault Systèmes Vélizy-Villacoublay, France).

### 2.9 Cultivation and age synchronization of C. elegans

The used *Caenorhabditis elegans* (*C. elegans)* strain wild-type Bristol N2, and its laboratory food source *E. coli* OP50 were obtained from the Caenorhabditis Genetics Center (CGC, University of Minnesota, MN, USA). Maintenance of the strain was performed as described previously on nematode growth medium (NGM) agar plates and cultivated at 20 °C [44]. Nematodes were age-synchronized by washing gravid adult worms off NGM plates with S Basal buffer containing 5 mg/ml PEG 3350 (Sigma Aldrich). The suspension was collected in a 15 ml reaction tube and the worms were settled by gravitation followed by another washing step with S Basal + PEG buffer to remove residual bacteria. Next, the nematodes were bleached with 1 % [v/v] alkaline hypochlorite solution, and the eggs released after ∼5 min of constant vortexing at room temperature. After centrifugation at 1300×*g* for 1 min, the eggs were washed twice with 5 ml S Basal + PEG buffer to remove residual bleaching solution.

Age-synchronized wild-type N2 nematodes were incubated on NGM plates at 20 °C for 48 h until they reached the L4 stage. They were fed OP50 heat-inactivated at 65 °C for 30 min and supplemented with either 50 μM of the treatment compounds, or no supplement (control group). Oxidative stress was induced using paraquat (PQ, Sigma Aldrich), a highly toxic compound known to increase ROS and cause mitochondrial dysfunction [45]. Approximately 1000 nematodes per group were transferred to NGM plates containing either 20 mM PQ to induce oxidative stress or no PQ for unstressed conditions. They were then incubated for 24 h until adulthood.

#### 2.9.1 Cellular stress analysis in C. elegans

For RNA isolation, nematodes were washed off plates using S Basal + PEG buffer and enzymatically lysed following a modified protocol [46] using Proteinase K (New England BioLabs, Ipswich, MA, USA). Total RNA was isolated using the RNeasy® Plus Kit (Qiagen, Hilden, Germany) according to the manufacturer's instructions. The acquired RNA concentration and ratio 260/280 nm were measured by POLARstar Omega microplate reader (BMG Labtech). For gene expression analysis, a total amount of 200 ng of the isolated RNA was first transcribed into cDNA using the iScript cDNA Synthesis Kit according to the manufacturer's instructions (Bio-Rad Laboratories). In detail, cDNA synthesis was carried out in a CFX Connect or CFX96 Real-Time System (Bio-Rad Laboratories) thermal cycler according to the following protocol: priming for 5 min at 25 °C, reverse transcription for 20 min at 46 °C and conclusively Reverse Transcriptase inactivation for 1 min at 95 °C. The obtained cDNA was diluted with DEPC-H_2_O to a final concentration of 2.5 ng/μl for subsequent gene expression analysis.

Then, mRNA expression levels of genes involved in oxidative stress were measured quantitatively by real-time quantitative polymerase chain reaction (RT-qPCR) using iQ SYBR Green Supermix (Bio-Rad Laboratories). RT-qPCR was performed by means of a thermal cycler starting with DNA denaturation and polymerase activation for 3 min at 95 °C followed by 40 amplification cycles consisting of denaturation for 10 s at 95 °C, primer annealing for 30 s at 57.5 °C and extension for 20 s at 72 °C. Finally, melt curve analysis was performed by gradually increasing the temperature from 65 °C to 95 °C in 0.5 °C steps.

Each experiment was performed in plate triplicate per day. Samples were measured in technical duplicate. The obtained gene expression data was normalized to multiple reference genes namely beta Actin (*act-1*), DNA-directed RNA polymerase II subunit RPB1 (*ama-1*) and Peroxisomal Membrane Protein-related protein (*pmp-3*). Target genes for *C. elegans* were fork head-related transcription factor (*daf-16*), glutamate-cysteine ligase (*gcs-1*), glutathione peroxidase 5 (*gpx-5*), glutathione S-transferase 4 (*gst-4*), protein skinhead-1 (*skn-1*) and mitochondrial superoxide dismutase [Mn] 2 (*sod-3*). Relative mRNA expression levels of the target genes were calculated using the 2^−ΔΔcT^ method [47]. Primers for oxidative stress were designed and evaluated according to MIQE guidelines [48]. Genes utilized in the subsequent experiments were selected based on literature research. Primers were ordered from Microsynth AG (Balgach, Switzerland) and their sequences are listed in Table 1.

**Table 1:** Oligonucleotide sequences of primers used for gene expression analysis in *C. elegans*.

Gene	Forward primer sequence (5’ → 3′)	Reverse primer sequence (5’ → 3′)	Accession number
Reference genes			
*act-1*	GTGTTCCCATCCATTGTC	GCTCATTGTAGAAGGTGTG	NM_073418.5
*ama-1*	CTCCGTCGTTGACTGTAT	ATACCCATTCCTCGTCTTC	NM_068122.6
*pmp-3*	ATACGAAGCCACGGATAG	CTGTGTCAATGTCGTGAAG	NM_001269679.1
Target genes			
*daf-16*	GAATGGATGGTCCAGAATG	GATTCCTTCCTGGCTTTG	AF032112.1
*gcs-1*	GATTCCCAGGTCTCATTTC	GCAGGATGAGATTGTACG	NM_063526.6
*gpx-5*	CGCTGGAGTCAATGTAAAG	GGGAAGGCAATGAGAGTA	NM_077214
*gst-4*	GTGCCTTACGAGGATTATAG	GTGATAGACATTGACTGACC	NM_069447
*skn-1*	GCAAGAGATGCGTGATTC	GTAGGCGTAGTTGGATGT	NM_171345.4
*sod-3*	GTGGTGGACACATCAATC	GCAATATCCCAACCATCC	NM_078363

### 2.10 Statistics

Statistical analysis was performed using Graphpad Prism version 10.1.0 (GraphPad Software, San Diego, CA, USA). If more than two groups were compared, the statistical difference among means was determined using an ordinary one-way ANOVA and Šídák's multiple comparison test or, if the standard deviations (SD) were significantly different, a Brown–Forsythe and Welch ANOVA test and Dunnett's multiple comparison test. For analysis of cytokine arrays, RStudio 2023.06.2 Build 561 (Posit PBC, Boston, MA, USA) with R version 4.3.1 (Vienna, Austria) was used. Normal distribution of samples was examined with a Shapiro-Wilk test, and homoscedasticity was confirmed with the Levene-test included in the car-package version 3.1–2. If both, normal distribution of samples and homoscedasticity could be confirmed, an analysis of variance model was used to identify significant differences between groups (α < 0.05). If heteroscedasticity was detected, a Welch-corrected analysis of variance model was applied. If two sample groups were compared, a student's t-test was applied, and *p*-values were adjusted with either the Benjamini-Hochberg or the Bonferroni (for significantly different signals caused by treatments per molecule) correction for multiply performed t-tests. Figures were prepared using CorelDRAW 2019 (Corel Corporation, Ottawa, ON, Canada).

## 3 Results

### 3.1 *Trans*-coumaroyl esterification enhances the cellular uptake and anti-inflammatory potential of TA

**Fig. 2:**
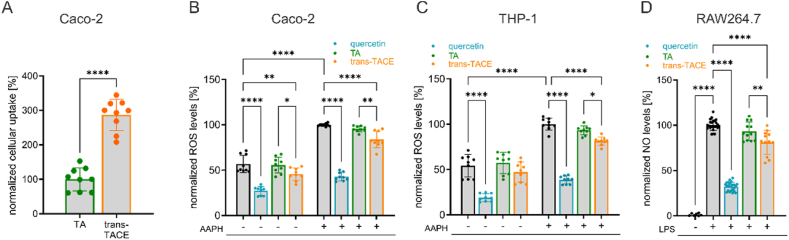
**Uptake efficiencies of TA and *trans*-TACE and their impact on ROS and NO levels in cellular models**. (A) Comparison of uptake efficiencies of TA and *trans*-TACE into differentiated Caco-2 cells after 4 h of treatment, determined via LC-MS after extraction, normalized to TA (*n* = 9). (B, C) Intracellular ROS levels in Caco-2 and THP-1 cells after a 20-min treatment with 10 μM TA, *trans*-TACE or quercetin and subsequent stress induction by 300 μM AAPH, normalized to stressor treatment (*n* = 9). (D) NO levels in the supernatant of RAW264.7 cells following a 24-h treatment with 10 μM TA, *trans*-TACE or 20 μM quercetin and 250 ng/ml LPS as the stress substance, normalized to the stressor treatment (*n* = 12–24). Data are presented as the means ± SD of 3–4 independent experiments. ∗*p* < 0.05; ∗∗*p* < 0.01; ∗∗∗∗*p* < 0.0001.

In this study, we aimed to compare the bioactivity of TA and its *trans*-hydroxycinnamoyl ester, *trans*-TACE. We first assessed the cellular uptake of TA and *trans*-TACE into differentiated Caco-2 cells, a widely used *in vitro* model that mimics the intestinal epithelium and serves as a predictor of intestinal absorption in humans [49]. Differentiated Caco-2 cells were treated with either TA or *trans*-TACE for 4 h, followed by cellular extraction and quantification of the compounds via LC-MS. As shown in Fig. 2A, *trans*-TACE exhibited a significantly enhanced cellular uptake, approximately three times higher than TA (*p* < 0.0001). This suggests that hydroxycinnamoyl esterification increases the intestinal permeability of TA, which may influence the compound's overall bioavailability and potential biological activity.

Next, we investigated the ability of TA and its 3-*O*-trans-*p*-coumaroyl ester *trans*-TACE to reduce ROS and NO levels under cellular stress conditions. Fig. 2B–C illustrate intracellular ROS levels in human Caco-2 and THP-1 cells, respectively, following treatment with TA or *trans*-TACE under both basal and AAPH-induced oxidative stress conditions. Additionally, quercetin was included as an antioxidant positive control. *Trans*-TACE significantly inhibited ROS production in both cell lines (*p* < 0.0001) under stress conditions and additionally decreased basal ROS levels in Caco-2 cells (*p* = 0.0068). In contrast, TA showed no significant effect on ROS levels in either Caco-2 or THP-1 cells, regardless of stress conditions. The difference between TA and *trans*-TACE was significant under both unstressed (*p* = 0.0202) and stressed (*p* = 0.0041) conditions in Caco-2 cells, as well as under stressed conditions in THP-1 cells (*p* = 0.0262).

Fig. 2D shows the percentages of NO levels in the supernatant of RAW264.7 macrophages following treatment with TA and *trans*-TACE in combination with LPS. A significant reduction in NO production was observed only with *trans*-TACE (*p* < 0.0001). *Trans*-TACE exhibited a significantly stronger reduction in NO levels compared to TA (*p* = 0.0013). NO levels in unstressed RAW264.7 cells were very low and remained unchanged upon treatment with TA or *trans*-TACE (see Supplementary Table 2). Taken together, these results indicate that *trans*-TACE is more effective than TA in reducing oxidative and nitrosative stress *in vitro*.

**Fig. 3:**
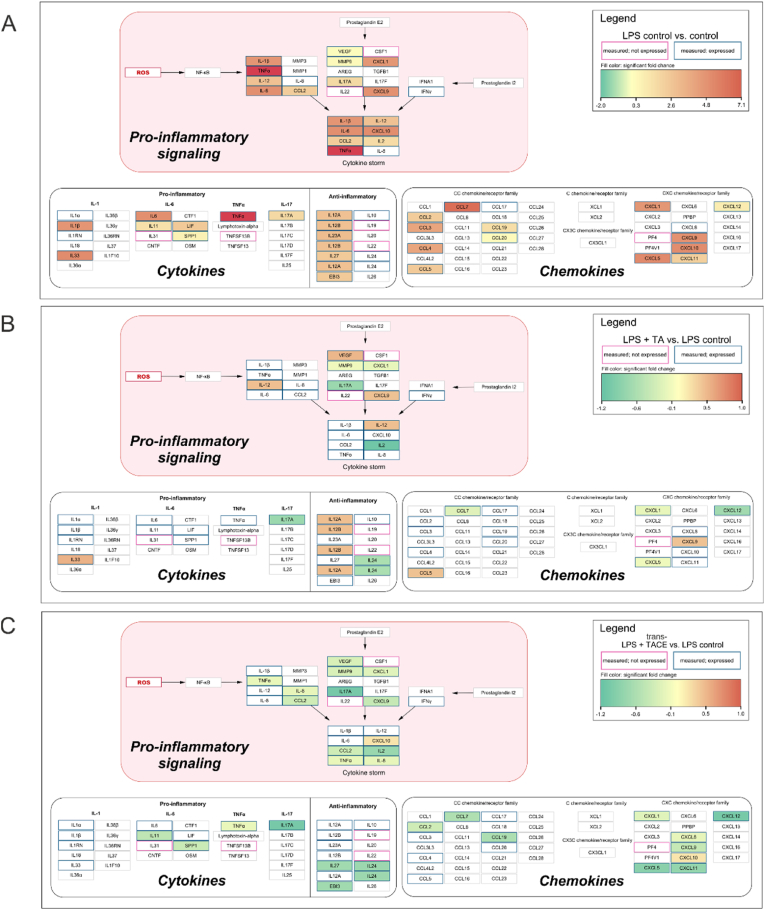
**Graphical overview of pro-inflammatory mediator expression profiles in THP-1 cells following LPS challenge and treatment with TA or trans-TACE.** (A) Fold changes in cytokine and chemokine levels between LPS treated cells and untreated control cells, quantified by cytokine array analysis. (B) Fold changes between LPS treatment and LPS treatment combined with 10 μM TA. (C) Fold changes between LPS treatment and LPS treatment combined with 10 μM *trans*-TACE. Cytokines and chemokines with pink borders were measured, but not expressed by the cells, while those with blue borders were both measured and expressed. Color-filled nodes represent significantly regulated targets (*p* < 0.05) and range from green (downregulation) to red (upregulation). All colored nodes indicate statistically significant modulation in response to treatment. Data are presented as the mean values of two independent experiments (*n* = 4).

To investigate the influence of TA and *trans*-TACE on pro-inflammatory signaling, we conducted a semi-quantitative cytokine array analysis. THP-1 macrophages were subjected to LPS to induce an inflammatory response and simultaneously treated with 10 μM TA or *trans*-TACE. Membranes pre-loaded with antibodies specific to 105 human cytokines and chemokines were incubated with the supernatants from the treated THP-1 cells. The captured analytes were visualized and semi-quantified as spot intensities (see Supplementary Tables 3 and 4). The overall expression profiles were summarized and visualized as fold changes in analyte spot intensities between the LPS treatment and control (Fig. 3A), LPS and TA (Fig. 3B), or LPS and *trans*-TACE (Fig. 3C) treatments. LPS stimulation resulted in the significant upregulation (visualized in red) of several pro-inflammatory cytokines and chemokines involved in NF-κB, prostaglandin E2, and I2 signaling pathways, ultimately leading to excessive pro-inflammatory cytokine release. As shown in Fig. 3B–C, additional treatment with TA or *trans*-TACE significantly reduced pro-inflammatory signaling by downregulating (visualized in green) the production and release of several pro-inflammatory mediators compared to LPS treatment alone. All significantly regulated cytokines in response to treatment are color-filled, ranging from green (downregulation) to red (upregulation). Notably, a broader panel of cytokines and chemokines was significantly downregulated following *trans*-TACE treatment, indicating a more pronounced anti-inflammatory effect compared to TA.

**Fig. 4:**
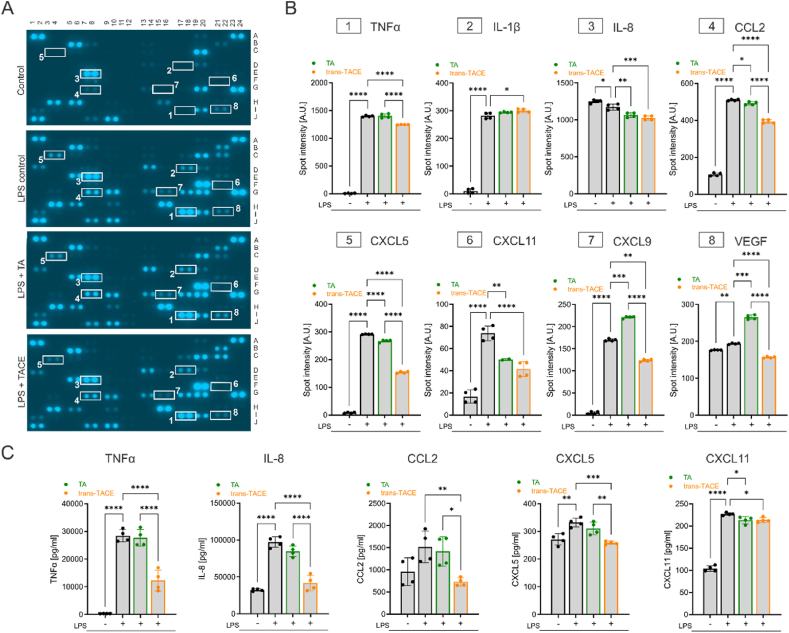
**Impact of TA and *trans*-TACE on the expression of selected cytokines and chemokines under LPS challenge.** (A) Representative images of cytokine array membranes incubated with supernatants of THP-1 cells treated with 10 μM TA or *trans*-TACE under LPS challenge. Cytokines and chemokines expressed are presented as duplicate spots and important analytes are framed and marked with numbers. (B) Corresponding spot intensities of the selected numbered analytes portrayed as arbitrary units (A.U.). (C) Concentrations of TNFα, IL-8, CCL2, CXCL5 and CXCL11 quantified by multiplex bead-based cytokine immunoassay. Data are presented as the means ± SD of 2 independent experiments (*n* = 4). ∗*p* < 0.05; ∗∗*p* < 0.01; *p*∗∗∗ <0.001; ∗∗∗∗*p* < 0.0001.

Fig. 4A displays representative images of the cytokine array membranes with visualized analyte spots. The spots corresponding to important analytes upregulated upon LPS challenge, compared to the control treatment, are framed and numbered. The corresponding spot intensity values of these targets are presented in Fig. 4B. Treatment with *trans*-TACE significantly reduced the production of several pro-inflammatory mediators, including TNFα, IL-8, CCL2, CXCL5, CXCL11, vascular endothelial growth factor (VEGF, all *p* < 0.0001) and CXCL9 (*p* = 0.0013), compared to the stressor. In contrast, TA treatment significantly inhibited the production of IL-8 (*p* = 0.0015), CCL2 (*p* = 0.0305), CXCL5 (*p* < 0.0001), and CXCL11 (*p* = 0.0048), but to a lesser extent. The differences in effects between TA and *trans*-TACE were highly significant for TNFα, CCL2, CXCL5, CXCL9 and VEGF. Notably, TA treatment resulted in significant increases in CXCL9 and VEGF levels, unlike *trans*-TACE. Additionally, *trans*-TACE slightly increased IL-1β levels (*p* = 0.0184) in the THP-1 cell supernatant, whereas TA exhibited no effect.

To further analyze and quantify the concentrations of TNFα, IL-8, CCL2, CXCL5, and CXCL11 in the supernatant of treated THP-1 macrophages, we employed a bead-based multiplex immunoassay (Fig. 4C). Consistent with the semi-quantitative results from the cytokine array analysis, *trans*-TACE treatment led to a significant decrease in the concentrations of TNFα (*p* < 0.0001), IL-8 (*p* < 0.0001), CCL2 (*p* = 0.0096), CXCL5 (*p* = 0.0003), and CXCL11 (*p* = 0.0330) as measured by the multiplex immunoassay. However, TA significantly reduced only the concentration of CXCL11 (*p* = 0.0295). The spot intensities for TNFα and IL-8 on the array membranes were near the detection limit, making treatment comparisons challenging, as confirmed by the more sensitive immunoassay analysis.

**Fig. 5:**
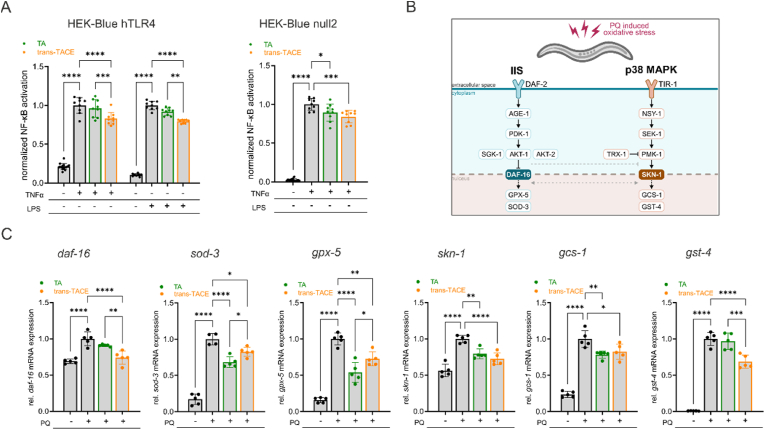
**Effects of TA and *trans*-TACE on NF-κB activation and oxidative stress-related gene expression in cellular and *C. elegans* models**. (A) NF-κB activation in TLR4-expressing HEK-Blue hTLR4 cells and non-TLR4-expressing HEK-Blue null2 cells following a 24-h treatment with TNFα (10 ng/ml), LPS (10 ng/ml) and TA or *trans*-TACE (5 μM), normalized to the stress control (*n* = 9). (B) Schematic overview of key signaling pathways regulating oxidative stress responses and immune defense in *C. elegans*. The IIS pathway regulates DAF-16 activity, controlling antioxidant and stress response genes (*sod-3, gpx-5*). The p38 MAPK pathway, activated via TIR-1, modulates SKN-1-dependent detoxification and immune defense genes (*gcs-1*, *gst-4*). Crosstalk between these pathways ensures coordinated stress adaptation. (C) Relative mRNA expression of *daf-16*, *sod-3*, *gpx-5*, *skn-1*, *gcs-1* and *gst-4* in *C. elegans* following a 24-h treatment with 50 μM TA or *trans*-TACE and stress induction by 20 mM PQ, normalized to PQ control (*n* = 5 biological, 4 technical replicates). Data are represented as means ± SD of 2–3 independent experiments × *p* < 0.05; ∗∗*p* < 0.01; ∗∗∗*p* < 0.001; ∗∗∗∗*p* < 0.0001.

To investigate whether the observed anti-inflammatory effects of TA and *trans*-TACE were mediated through targeting the central transcription factor NF-κB via the LPS-activated receptor TLR4 or via a TLR4-independent pathway, we used the TLR4 reporter cell line HEK-Blue hTLR4, which stably co-expresses the human *TLR4* gene and SEAP, along with its parental cell line HEK-Blue null2, which only expresses SEAP. The production and secretion of active SEAP directly correlate with the activation of NF-κB. Fig. 5A shows the levels of NF-κB activation measured in the supernatant of HEK-Blue hTLR4 and HEK-Blue null2 cells following treatment with the stressors TNFα or LPS and the compounds TA and *trans*-TACE. NF-κB activation significantly increased with the addition of TNFα and LPS to HEK-Blue hTLR4 cells, whereas only TNFα led to an increase in NF-κB activation in HEK-Blue null2 cells. This was expected due to the absence of TLR4 in HEK-Blue null2 cells, rendering them unresponsive to LPS (see Supplementary Table 5). Treatment with 5 μM *trans*-TACE significantly inhibited both TNFα- (*p* < 0.0001) and LPS-induced (*p* < 0.0001) NF-κB activation in HEK-Blue hTLR4 cells, as well as TNFα-induced NF-κB activation in HEK-Blue null2 cells (*p* = 0.0002). In contrast, TA at 5 μM only slightly reduced TNFα-induced NF-κB activation in HEK-Blue null2 cells (*p* = 0.0166). Basal NF-κB levels were very low in unstressed cells and remained unaffected by treatment with either compound (see Supplementary Table 5). These results suggest that *trans*-TACE inhibits both TLR4-dependent and TLR4-independent NF-κB activation, whereas TA shows only a slight tendency to reduce active NF-κB levels.

In summary, these data suggest that the TA derivate exhibits higher *in vitro* anti-inflammatory properties compared to its non-conjugated parent compound, as evidenced by a more substantial reduction in pro-inflammatory signaling mediators under stress conditions as a result of decreased NF-κB activation.

To validate these findings in an *in vivo* model, we examined the impact of TA and *trans*-TACE on stress-related gene expression in *C. elegans*, a well-established system for studying oxidative stress and immune regulation. This model allows for a systems biology approach, enabling the assessment of whole-organism responses and providing insights into the molecular mechanisms underlying the effects of hydroxycinnamoyl esterification on stress adaptation. Fig. 5B illustrates the analyzed genes which are regulated by two major pathways involved in oxidative stress defense and immune regulation, although other signaling cascades also contribute. The insulin and insulin-like growth factor signaling (IIS) pathway, activated by DAF-2, regulates the forkhead box O (FOXO) transcription factor DAF-16. Under stress, inhibition of IIS allows DAF-16 nuclear translocation, inducing genes related to oxidative stress resistance (*sod-3*, *gpx-5*) and immune defense. The p38 MAPK pathway, initiated by the toll/interleukin-1 receptor (TIR)-domain protein TIR-1, activates SKN-1, which regulates detoxification and immune-related genes (*gcs-1*, *gst-4*). The p38 MAPK pathway is also crucial for host defense, especially against bacterial infections. Importantly, IIS and p38 MAPK pathways exhibit crosstalk, with both pathways jointly influencing stress responses [50]. As shown in Fig. 5C, exposure of *C. elegans* to PQ for 24 h significantly upregulated stress response genes, including *daf-16, sod-3, gpx-5, skn-1, gcs-1,* and *gst-4* (*p* < 0.0001). Treatment of the worms with 50 μM *trans*-TACE significantly reduced PQ-induced expression of all analyzed target genes, specifically *daf-16* (*p* < 0.0001), *gst-4* (*p* < 0.0001), *sod-3* (*p* = 0.0113), *gpx-5* (*p* = 0.0013), *skn-1* (*p* < 0.0001)*,* and *gcs-1* (*p* = 0.0198). TA treatment similarly reduced gene expression of *skn-1* (*p* = 0.0018) and *gcs-1* (*p* = 0.0039), while it showed no effect on *daf-16* and *gst-4*. Treatment with TA led to a slightly higher reduction in *gpx-5* and *sod-3* gene expression compared to *trans*-TACE.

Overall, these findings indicate that *trans*-TACE has a broader protective effect than TA, significantly reducing all tested genes involved in stress response in *C. elegans*. This aligns with our *in vitro* results, where *trans*-TACE showed a stronger reduction in pro-inflammatory cytokines, indicating that its broader impact on both oxidative stress and inflammation may make it a more potent compound for mitigating cellular stress pathways.

### 3.2 NF-κB signaling modulation by *trans*-TACE is isoform-specific

**Fig. 6:**
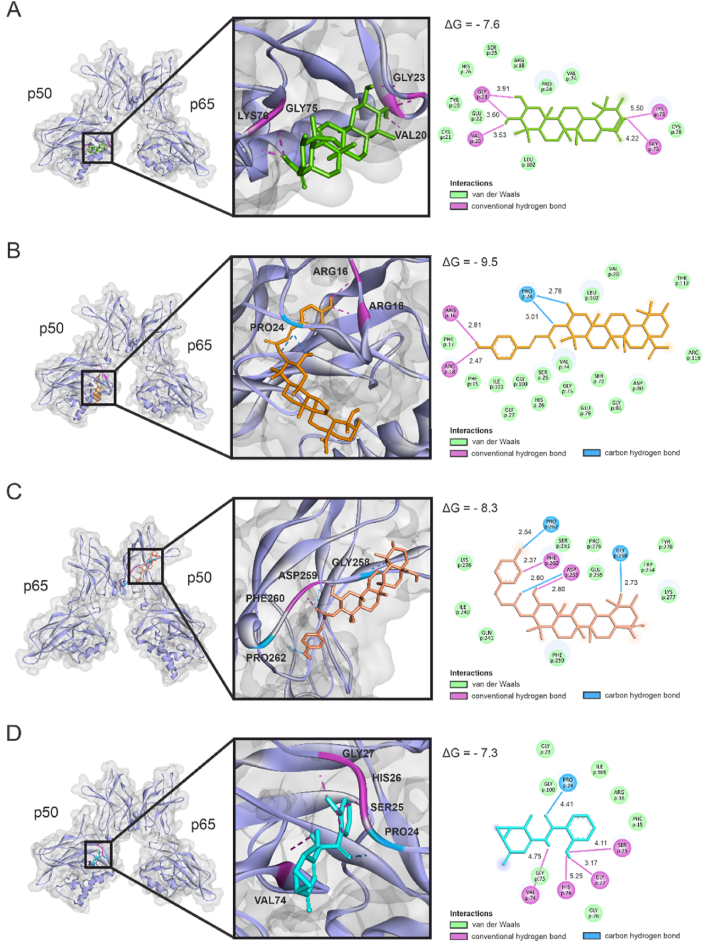
**Molecular docking simulations of TA, *trans*-TACE, *cis*-TACE and DHMEQ with NF-κB (p50/p65).** The three-dimensional diagrams illustrate the predicted binding conformations of TA (A), *trans*-TACE (B), *cis*-TACE (C) and DHMEQ (D) with NF-κB (p50/p65), representing the lowest estimated free energy change (ΔG) upon binding. Two-dimensional diagrams display amino acid residues of NF-κB potentially involved in interactions with each ligand. Predicted distances for each binding interaction are labeled and provided in Ångströms (Å).

To gain deeper insights into the molecular basis and structural impact of hydroxycinnamoyl esterification and geometric isomerism, we performed molecular docking simulations with the NF-κB (p50/p65) complex, comparing TA with both *trans*- and *cis*-TACE. Molecular docking enables the prediction of ligand binding affinity and interaction sites, providing insights into potential interference with NF-κB signaling. The known NF-κB inhibitor, DHMEQ, was included as a reference due to its established direct inhibitory action on NF-κB [38]. Molecular docking results highlighted distinct binding patterns and affinities of TA (Fig. 6A), the trans isoform of TACE (Fig. 6B), the cis isoform of TACE (Fig. 6C) and DHMEQ (Fig. 6D), with NF-κB (p50/p65), focusing on the p50 subunit. The trans isoform of TACE was predicted to bind within the DNA-binding site of p50, exhibiting a higher affinity (ΔG = −9.5) compared to DHMEQ (ΔG = −7.3). It formed a carbon hydrogen bond with PRO24 and hydrogen bonds with ARG16 and ARG18, both of which are key residues that directly interact with DNA. These residues have interface residue buried fractions (IRBFs) of 0.69 and 0.60 with the DNA chain, respectively, indicating their significant role in DNA binding. The IRBF represents the proportion of a residue's surface area that becomes shielded upon DNA binding, with higher values indicating stronger involvement in the interaction [39]. In comparison, DHMEQ was also predicted to bind in the DNA-binding region of p50, though with differing specific interactions. It also established a carbon hydrogen bond with PRO24 and additionally formed hydrogen bonds with VAL74, SER25, HIS26 and GLY27. Among these, SER25, HIS26, and GLY27 are directly involved in DNA binding, with IRBFs of 0.36, 0.72 and 0.6, respectively. TA was predicted to bind at a similar site (ΔG = −7.6) and established hydrogen bonds with VAL20, GLY23, GLY75 and LYS76 with only the latter displaying a low IRBF of 0.01 with the DNA chain. The cis isoform of TACE, in contrast, bound outside the DNA-binding domain of p50, showing a similar affinity (ΔG = −8.3). *Cis*-TACE's binding involved conventional hydrogen bonds with PHE260 and ASP259 and carbon hydrogen bonds with PRO262 and GLY256, neither of which are involved in DNA binding. These findings suggest that the trans isoform of TACE may interfere with p50-DNA interactions by targeting these critical residues.

Following the molecular docking findings, which revealed distinct binding differences between the cis and trans isoforms, we aimed to determine whether these structural variations influence the bioactivity of TACE. To address this, we compared the effects of pure *cis*-TACE with a trans/*cis*-TACE mixture in cellular models and *C. elegans*, aiming to clarify the functional relevance of TACE's geometric isomerism.

**Fig. 7:**
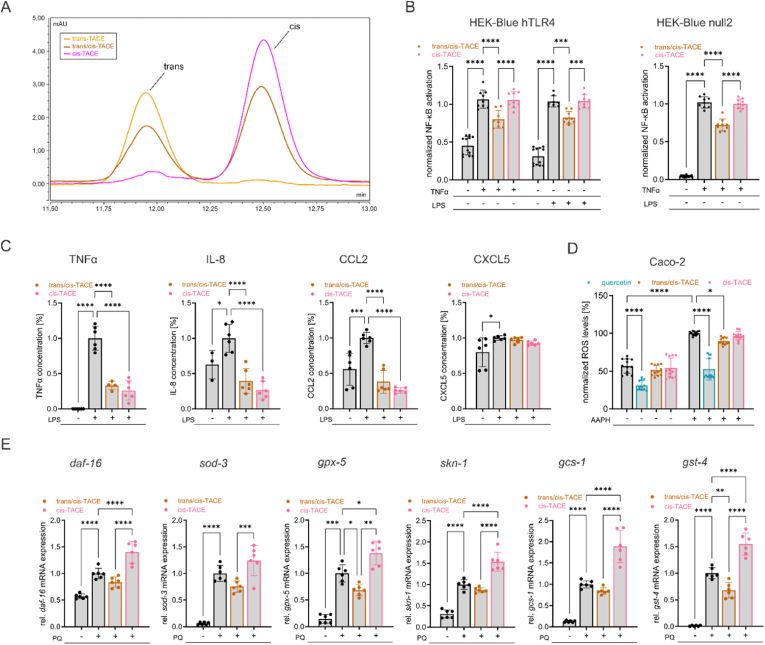
**Comparative effects of trans/*cis*-TACE and *cis*-TACE on key targets of oxidative and inflammatory stress in cellular and *C. elegans* models.** (A) LC-MS chromatograms comparing the cis and trans isoform content of *trans*-TACE, trans/*cis*-TACE and *cis*-TACE. (B) NF-κB activation in TLR4-expressing HEK-Blue hTLR4 cells and non-TLR4-expressing HEK-Blue null2 cells following a 24-h treatment with TNFα (10 ng/ml), LPS (10 ng/ml) and trans/*cis*-TACE or *cis*-TACE (5 μM), normalized to the stress control (*n* = 9–12). (C) Concentrations of TNFα, IL-8, CCL2 and CXCL5 in LPS (250 ng/ml)-stimulated THP-1 cells following a 4-h treatment with 10 μM trans/*cis*-TACE or *cis*-TACE, quantified by multiplex bead-based cytokine immunoassay (*n* = 4–6). (D) Intracellular ROS levels in Caco-2 cells after 20 min of treatment with 10 μM trans/*cis*-TACE, *cis*-TACE or quercetin and subsequent stress induction by 300 μM AAPH, normalized to stressor treatment (*n* = 12). (E) Relative mRNA expression of *daf-16*, *gst-4*, *gpx-5*, *skn-1*, *gcs-1* and *sod-3* in *C. elegans* following a 24-h treatment with 50 μM trans/*cis*-TACE or *cis*-TACE and stress induction by 20 mM PQ, normalized to PQ control (*n* = 6 biological, 4 technical replicates). Data are represented as means ± SD of 2–3 independent experiments × *p* < 0.05; ∗∗*p* < 0.01; ∗∗∗*p* < 0.001; ∗∗∗∗*p* < 0.0001.

First, we analyzed the cis/trans ratios of *trans*-TACE, the trans/*cis*-TACE mixture, and *cis*-TACE via LC-MS (Fig. 7A). We identified a single dominant peak corresponding to the trans isoform in *trans*-TACE, a single peak for the cis isoform in *cis*-TACE, and two peaks in trans/*cis*-TACE, corresponding to both the trans and cis isoforms. Notably, trans/*cis*-TACE displayed a larger peak for the cis isoform, revealing a trans/cis ratio of approximately 4:6.

To compare the effects of trans/*cis*-TACE and *cis*-TACE on NF-κB activation, we applied the same NF-κB reporter assay used for TA and *trans*-TACE. As shown in Fig. 7, Fig. 5 μM of trans/*cis*-TACE strongly inhibited TNFα- (*p* < 0.0001) and LPS-induced (*p* = 0.0007) NF-κB activation in HEK-Blue hTLR4 cells and TNFα-induced NF-κB activation in HEK-Blue null2 cells (*p* < 0.0001). This indicates that trans/*cis*-TACE inhibits both TLR4-dependent and TLR4-independent NF-κB activation, similarly to the pure *trans*-TACE sample. To further validate the assay and provide a reference for comparison, we additionally tested the known NF-κB inhibitor DHMEQ, which showed similarly strong inhibition across both cell lines (Supplementary Fig. S1). In contrast, *cis*-TACE at the same concentration did not significantly alter NF-κB activation. The differences between trans/*cis*-TACE and *cis*-TACE were statistically significant under all tested conditions.

Next, we examined the effects of 10 μM trans/*cis*-TACE and *cis*-TACE on key pro-inflammatory mediators in LPS-activated THP-1 macrophages by employing a bead-based multiplex immunoassay. As shown in Fig. 7C, both the trans/*cis*-TACE mixture and the pure *cis*-TACE significantly downregulated the concentration of TNFα, IL-8 and CCL2 under LPS-induced stress (*p* < 0.0001), while CXCL5 concentrations remained unaffected by this treatment.

Additionally, we evaluated the ability of trans/*cis*-TACE and *cis*-TACE to reduce ROS levels under oxidative stress. As shown in Fig. 7C, intracellular ROS levels in human Caco-2 cells were measured following treatment with trans/*cis*-TACE or *cis*-TACE and the stressor AAPH. Treatment with trans/*cis*-TACE at 10 μM significantly reduced ROS levels under stress conditions (*p* = 0.039), whereas *cis*-TACE had no significant effect. Neither compound altered basal ROS levels under unstressed conditions. These findings suggest that the antioxidant effects observed are primarily mediated by the *trans*-isoform, as the pure cis isoform was inactive. The trans/*cis*-TACE mixture showed similar ROS scavenging effects compared to the pure *trans*-TACE.

Overall, these findings strongly indicate that the trans isoform of TACE is essential for its bioactivity. To validate these observations *in vitro*, we conducted the same cellular stress-related gene expression assay in *C. elegans* as previously performed. Co-treatment with trans/*cis*-TACE attenuated the PQ-induced gene expression of the analyzed targets, showing a general trend toward downregulation, with significant reductions in *gpx-5* (*p* = 0.0282) and *gst-4* (*p* = 0.0024). In contrast, treatment with *cis*-TACE further amplified the PQ-induced expression of *daf-16* (*p* < 0.0001), *gpx-5* (*p* = 0.0282), *skn-1* (*p* < 0.0001), *gcs-1* (*p* < 0.0001), and *gst-4* (*p* = 0.0024). The differences between the effects of trans/*cis*-TACE and *cis*-TACE were significant for all analyzed genes. These results suggest that while the cis isoform of TACE plays a role in modulating pro-inflammation cytokines *in vitro*, the trans isoform, whether alone or in mixture, exerts a more robust protective effect under oxidative and inflammatory stress both *in vitro* and *in vivo*, highlighting the importance of isomer-specific effects in stress adaptation and immune regulation.

## 4 Discussion

In this study, we aimed to investigate how the 3-*O*-*p*-coumaroyl ester modification of TA changes its biological properties. We compared the efficacy of TA and *trans*-TACE in mitigating oxidative stress and reducing pro-inflammatory responses, using both *in vitro* and *in vivo* models. Our results highlight the significant impact of 3-*O*-*p*-coumaroyl esterification on enhancing the bioactivity and bioavailability of TA.

First, to analyze the impact of the ester modification on the bioavailability of pentacyclic triterpenes, we conducted cellular uptake tests with TA and *trans*-TACE using differentiated Caco-2 cells. These cells express several morphological and functional characteristics of mature enterocytes, making them well-suited for absorption and uptake studies [49]. Plant bioactive compounds often exhibit low bioavailability, limiting their bioactive properties [51]. Our findings revealed that *trans*-TACE exhibited approximately threefold higher uptake efficiency into differentiated Caco-2 cells compared to TA. Importantly, there is currently a lack of data on the bioavailability of TA and *trans*-TACE in cellular models, underscoring the necessity for further investigation into their cell penetration mechanisms. Research on the bioavailability of pentacyclic triterpenes generally indicates low and variable uptake and transport rates among different derivatives, suggesting differences in their absorption dynamics [52]. The enhanced uptake efficiency of *trans*-TACE observed in our study implies that the 3-*O*-trans-*p*-coumaroyl ester modification improves the cellular permeability of TA, potentially enhancing its bioavailability and biological activity. This increased uptake may be attributed to structural modifications that influence lipophilicity, membrane interactions, or transporter affinities [53]. Given the generally poor bioavailability of pentacyclic triterpenes, our findings highlight the potential of esterification as a strategy to improve cellular absorption.

We further focused on the potential antioxidant properties of TA and *trans*-TACE. Since excessive levels of ROS and NO contribute to oxidative stress and inflammation, which play a key role in chronic disease pathogenesis, we sought to evaluate the antioxidant potential of these compounds [1]. Bioactive compounds from plants, such as quercetin, have been demonstrated to mitigate oxidative stress and inflammation in cellular models by scavenging free radicals and modulating inflammatory pathways [54,55]. In this study, we also demonstrated that *trans*-TACE significantly reduced levels of ROS in Caco-2 and THP-1 cells, as well as NO in RAW264.7 cells under stress conditions. Conversely, TA exhibited minimal to no inhibitory effects on ROS and NO production, consistent with previous reports indicating TA's limited radical scavenging activity in cell-free *in vitro* assays [12,13,56]. Previous studies have highlighted TA's capability to mitigate hydrogen peroxide-induced ROS generation and reduce inducible nitric oxide synthase expression in various cellular models, including rat vascular smooth muscle cells [57] and neuronal cells under neurotoxic conditions [58]. Additionally, TA has shown activity in reducing NO levels in LPS-stimulated RAW264.7 cells [59]. In our study, we found that *trans*-TACE exhibited significantly higher inhibitory activity against ROS production in THP-1 macrophages and NO production in RAW264.7 cells compared to TA. Currently, data on the cellular antioxidant activity of *trans*-TACE remains limited, although studies with *trans*-TACE isolated from *Ligustrum robustum* have reported superoxide and hydroxyl radical scavenging properties in cell-free analyses [60]. The observed reduction in NO levels by *trans*-TACE aligns with findings by Cheng et al., who reported inhibitory effects of *trans*-TACE on NO production in RAW264.7 macrophages [18]. To our knowledge, this is the first study to directly compare the antioxidant capabilities of TA and *trans*-TACE in cellular models, revealing that hydroxycinnamoyl esterification enhances TA's ability to inhibit ROS and NO production. Importantly, quercetin was included as a positive control to verify the performance responsiveness of the ROS assay, given it's well-documented and potent antioxidant properties [54]. However, it is worth noting that the commonly used H_2_DCFDA-based assay has limited specificity and sensitivity for ROS and is known to produce artifacts under certain cellular conditions [61]. Therefore, the weak effects of TA and *trans*-TACE observed *in vitro* should be interpreted with caution.

Next, we conducted an analysis of pro-inflammatory cytokine and chemokine expression profiles in LPS-stimulated THP-1 cells following treatment with TA and *trans*-TACE using cytokine array analysis. LPS stimulation markedly upregulated the production of numerous pro-inflammatory markers implicated in NF-κB, prostaglandin E2, and I2 signaling pathways. While these findings align with existing literature [[62], [63], [64]], this study represents the first comprehensive demonstration of this effect across a broad spectrum of chemokines and cytokines. Treatment of cells with TA and *trans*-TACE resulted in distinct protein expression profiles characterized by reduced levels of pro-inflammatory markers, with *trans*-TACE exhibiting a more potent inhibitory effect compared to TA. To complement these semi-quantitative measurements, we conducted bead-based multiplex immunoassay analyses targeting key cytokines and chemokines. Specifically, TA significantly decreased the concentration of CXCL11, whereas *trans*-TACE significantly reduced the levels of TNFα, IL-8, CCL2, CXCL5, and CXCL11 in the supernatant of treated THP-1 macrophages. The anti-inflammatory activity of TA has been documented in the literature. Previous studies have reported its ability to mitigate LPS-induced IL-8 in human gingival fibroblasts [65], and TNFα and IL-1β in BV2 microglia cells [66] at concentrations ranging from 15 to 60 μM. Results of the cytokine array indicated a slight reduction in IL-8 levels by TA, which was not consistently observed in the immunoassay. Furthermore, TA did not exhibit inhibitory effects on TNFα in either assay, consistent with findings by Choi et al. [67], where TA isolated from *Prunella vulgaris* did not alter TNFα levels in human mast cells, compared to the stressor treatment. Banno et al. [27] described anti-inflammatory activity of *trans*-TACE against 12-*O*-tetradecanoylphorbol-13-acetate (TPA)-induced inflammation in mice, however, limited data are available regarding its impact on inflammatory processes beyond this context.

To better understand the mechanisms behind the inhibitory effects of TA and *trans*-TACE on pro-inflammatory signaling, we examined their impact on TLR4 and NF-κB activation using HEK-Blue reporter cells. *Trans*-TACE significantly reduced NF-κB activation induced by both TNFα and LPS. By utilizing two different cell lines, including one stably expressing TLR4, we demonstrated that *trans*-TACE inhibited NF-κB activation through both TLR4-dependent and TLR4-independent pathways. In contrast, TA showed limited effectiveness in these assays. Previous studies have reported that TA can suppress IL-1β-induced inflammatory responses by inhibiting the NF-κB pathway in human osteoarthritic chondrocytes [68] and also reduce LPS-induced NF-κB activation in BV2 microglia cells [66] and human gingival fibroblasts [65], with a focus on TLR4-dependent mechanisms. However, these studies employed different assays and did not distinguish between TLR4-dependent and -independent activation. Our study is the first to show that *trans*-TACE not only exhibits stronger inhibitory effects on both TLR4-mediated and non-TLR4-mediated NF-κB activation compared to TA but also introduces a novel approach by differentiating the two modes of NF-κB activation. This distinction underscores the enhanced efficacy of *trans*-TACE, potentially due to its structural modifications.

Pentacyclic triterpenes and their derivatives in general have been widely recognized for their ability to influence inflammatory pathways, particularly by inhibiting NF-κB signaling. A study by Patil et al. demonstrated that pentacyclic triterpenes, such as corosolic acid or ursolic acid, can suppress IκB kinase-β (IKKβ), a key kinase involved in NF-κB activation [69]. Since NF-κB is highly responsive to oxidative stress, ROS can serve as secondary messengers that activate this pathway, further amplifying inflammatory responses. Conversely, inflammation itself can increase ROS production, creating a self-sustaining cycle of oxidative damage and immune activation [70]. Given that *trans*-TACE reduced intracellular ROS levels, albeit less effectively than quercetin, it is likely that its primary mode of action is not direct radical scavenging. This is consistent with previous studies indicating that pentacyclic triterpenes not only inhibit inflammatory mediators but can also enhance cellular antioxidant defenses through the regulation of key detoxification and redox-balancing pathways [71,72]. These findings raise the possibility that TACE influences multiple antioxidant defense mechanisms. This interpretation is further supported by the limited sensitivity of the DCFDA-based ROS assay, which may not detect more subtle intracellular changes in ROS. To obtain mechanistic insight beyond direct ROS detection, we extended our analyses to the *in vivo* model *C. elegans*, where oxidative stress responses can be assessed at the transcriptional level. *C. elegans* was chosen for this study due to its well-characterized genetic background, short lifespan, and the conserved pathways involved in oxidative and inflammatory stress responses, making it an ideal model for evaluating the potential therapeutic effects of TA and *trans*-TACE in a complex biological system [73]. Two important pathways involved in regulating stress response in *C. elegans* are the IIS pathway and the p38 MAPK pathway, both of which play critical roles in maintaining cellular homeostasis and stress resistance [50]. The IIS pathway is initiated by the DAF-2 receptor, a homolog of the human insulin and insulin-like growth factor (IGF)-1 receptors, which regulates stress responses, metabolism, and longevity through the transcription factor DAF-16, a member of the FOXO family. Under normal conditions, IIS activation leads to the phosphorylation of DAF-16 by AKT kinases, keeping it sequestered in the cytoplasm. However, under stress conditions or reduced IIS activity, DAF-16 translocates into the nucleus and induces the expression of genes involved in oxidative stress defense, detoxification, and immune responses, including *gpx-5* and *sod-3* [74]. In parallel, the p38 MAPK pathway, initiated by TIR-1, plays a key role in the innate immune response of *C. elegans*. Although *C. elegans* lacks a canonical NF-κB homolog and the classical MyD88–IKK–NF-κB signaling cascade found in mammals, its immune responses are orchestrated by conserved MAPK signaling networks. The NSY-1–SEK-1–PMK-1 module represents a core inflammatory signaling axis that coordinates transcriptional responses to microbial challenge and environmental stress [50]. This pathway has been shown to control the expression of numerous immune effector genes and functionally mirrors key aspects of mammalian inflammatory signaling.

In addition, PMK-1 signaling regulates the transcription factor SKN-1, the *C. elegans* ortholog of mammalian nuclear factor erythroid 2-related factor 2 (NRF2), which governs oxidative stress responses through transcriptional activation of genes such as *gst-4*, *gcs-1*, and *gpx-5* [75]. However, the IIS and p38 MAPK pathways are not strictly independent, nor do they function in isolation. Instead, they exhibit significant crosstalk, particularly in the regulation of stress responses and immunity, reflecting the intricate and highly coordinated nature of *C. elegans* stress adaptation mechanisms [76].

Here, we assessed the effects of *trans*-TACE and TA on cellular stress-related gene expression in *C. elegans* under paraquat-induced stress. Both compounds reduced the expression of key target genes; however, *trans*-TACE demonstrated a broader protective effect by downregulating a wider range of stress-responsive genes, including *daf-16* and *gst-4*, which remained unaffected by TA. Given that DAF-16 is a central regulator of the IIS pathway and *gst-4* encodes a glutathione S-transferase (GST) critical for detoxifying oxidative stress-induced lipid peroxidation products and xenobiotics [77,78], these findings suggest that *trans*-TACE more strongly modulates key cellular defense pathways. The observed reduction in *skn-1*, *gst-4*, and *gpx-5* further indicates suppression of redox-sensitive transcriptional programs linked to the p38 MAPK–SKN-1 axis. Together, these findings suggest that *trans*-TACE attenuates the oxidative stress response through coordinated modulation of conserved stress-adaptive mechanisms. This distinction aligns with our *in vitro* findings, where *trans*-TACE more effectively inhibited NF-κB activation, ROS and pro-inflammatory cytokine production compared to TA. The modulation of both pro-inflammatory and oxidative stress response genes observed in our *C. elegans* experiments supports the idea that *trans*-TACE influences conserved immune signaling pathways with functional similarity to mammalian NF-κB-dependent responses.

While most studies on TACE have predominantly focused on the trans isoform [[25], [26], [27]], it naturally occurs as a mixture of cis and trans isomers. Since even subtle structural variations can influence biological activity by altering molecular interactions, target binding or stability [[79], [80], [81]], we further aimed to assess the impact of geometric isomerism on TACE bioactivity. We first explored the potential of TA, *trans*-TACE and *cis*-TACE as direct NF-κB inhibitors by investigating their binding interactions through molecular docking simulations. The molecular docking results align with previous studies on inhibitors predicted to target the DNA-binding region of the p50 subunit of NF-κB, supporting *trans*-TACE's potential mechanism of action. For example, andrographolide, a labdane diterpenoid from *Andrographis paniculata*, is predicted to bind to the same DNA-interaction region of p50 as the trans isoform of TACE and the inhibitor DHMEQ, effectively blocking NF-κB activation in HEK293 cells using a similar SEAP reporter assay, a result we mirrored in our HEK-Blue reporter assay [82]. This suggests that occupying this region disrupts NF-κB's DNA binding capacity, thereby modulating downstream transcriptional activity. Additionally, compounds from *Blumea lanceolaria* essential oil are also predicted to interact with overlapping residues on p50, including PRO24 and ARG18 with the trans isoform of TACE, and VAL74, PRO24, HIS26 and SER25 with DHMEQ. This further corroborates that binding to these residues may suppress pro-inflammatory mediators such as TNFα and inducible nitric oxide synthase (iNOS) in RAW264.7 cells [83]. In contrast, the cis isoform of TACE was predicted to bind to a distinct region on p50, which does not directly impact the DNA-binding domain.

To further explore these differences, we examined the effects of pure *cis*-TACE and a trans/*cis*-TACE mixture on TLR4 and NFκB activation using our HEK Blue reporter cells. Consistent with our previous findings on pure *trans*-TACE, the trans/*cis*-TACE mixture effectively inhibited NFκB activation through both TLR4 dependent and TLR4 independent pathways. In contrast, pure *cis*-TACE showed no effect on NFκB activation. Interestingly, while the trans/*cis*-TACE mixture significantly reduced the production of key proinflammatory cytokines, showing strong inhibitory effects similar to pure *trans*-TACE, the pure cis isoform also demonstrated potent cytokine suppression, indicating the involvement of an alternative, NF-κB-independent mechanism. However, unlike *trans*-TACE, *cis*-TACE did not inhibit ROS production, suggesting that its mechanism of action is independent of both NFκB and oxidative stress modulation. These observed differences in NFκB inhibition align with our molecular docking findings, reinforcing the idea that structural variations arising from geometric isomerism influence the biological activity of TACE. Moreover, in *C. elegans*, the trans/*cis*-TACE mixture downregulated stress response genes in a manner similar to pure *trans*-TACE, likely through modulation of both the IIS and p38 MAPK pathways. In contrast, treatment with pure *cis*-TACE led to a marked increase in stress related gene expression, suggesting that rather than mitigating oxidative stress, it may have exacerbated it. The observed upregulation of *gst-4* and *gcs-1*, both downstream targets of SKN-1, indicates enhanced activation of this stress responsive pathway, which is typically associated with increased cellular stress [84].

These findings highlight the importance of considering isomer specific effects when evaluating the therapeutic potential of hydroxycinnamoyl esters of pentacyclic triterpenes. While the trans isoform appears to exert broad anti-inflammatory and cytoprotective effects by targeting NF-κB signaling, the cis isoform may engage distinct alternative pathways with potentially different physiological implications. There is strong indication that additional, yet unidentified pathways contribute to the biological effects of both *cis*- and *trans*-TACE.

To summarize, we demonstrated that the 3-*O*-trans-*p*-coumaroyl ester derivative of TA, *trans*-TACE, shows significantly enhanced ability to combat oxidative stress and inhibit pro-inflammatory signaling *in vitro*, primarily due to more effective inhibition of NF-κB activation and improved cellular uptake. In *C. elegans*, *trans*-TACE facilitated broader modulation of cellular defense mechanisms, particularly those linked to glutathione metabolism and immune regulation. Notably, we highlighted the isomer-specific antioxidant and anti-inflammatory activities of TACE, both *in vitro* and *in vivo*. This novel discovery enhances our understanding of *trans*-TACE's mode of action and underscores the importance of isoform-specific activity in its therapeutic potential.

## 5 Conclusions

Based on the comprehensive *in vitro* and *in vivo* analyses conducted in this study, our findings underscore the significant impact of chemical modification on the biological activity of pentacyclic triterpenes. By comparing TA and its 3-*O*-trans-*p*-coumaroyl ester derivative, *trans*-TACE, we demonstrated that *trans*-TACE exhibits enhanced anti-inflammatory properties, primarily through the modulation of the NF-κB signaling pathway. While we did not investigate specific post-translational modifications or protein alterations, our functional assays demonstrate significant changes in NF-κB activity, which correlate with the observed anti-inflammatory effects. In *C. elegans*, *trans*-TACE modulated a broader range of stress response mechanisms and strongly downregulated key stress related genes, suggesting a more comprehensive cellular defense against oxidative damage and inflammation. Notably, our results highlight the isoform specific bioactivity of TACE and functional significance of the trans isoform as the main bioactive form. These findings provide valuable insights into the potential of chemical modifications to optimize the bioactivity of natural compounds and offer a foundation for further research into the therapeutic potential of *trans*-TACE. Future studies should investigate the bioavailability, metabolic fate, and long-term effects of *trans*-TACE and TA in mammalian models to deepen our understanding of their mechanisms of action and their therapeutic applications.

## CRediT authorship contribution statement

**Mara Heckmann:** Conceptualization, Data curation, Investigation, Methodology, Writing – original draft, Writing – review & editing. **Lea Karlsberger:** Investigation, Methodology. **Bernhard Blank-Landeshammer:** Data curation, Investigation, Methodology. **Gerald Klanert:** Data curation, Methodology. **Nadiia Sadova:** Investigation, Methodology. **Verena Stadlbauer:** Data curation, Methodology. **Georg Sandner:** Data curation, Methodology. **Theresa Gramatte:** Data curation, Investigation. **Simone Kasis:** Data curation. **Julian Weghuber:** Conceptualization, Funding acquisition, Project administration.

## Data availability statement

The data are available from the authors upon request.

## Declaration of competing interest

The authors declare that they have no known competing financial interests or personal relationships that could have appeared to influence the work reported in this paper.
